# Light, heat, action: neural control of fruit fly behaviour

**DOI:** 10.1098/rstb.2014.0211

**Published:** 2015-09-19

**Authors:** David Owald, Suewei Lin, Scott Waddell

**Affiliations:** Centre for Neural Circuits and Behaviour, University of Oxford, Tinsley Building, Mansfield Road, Oxford OX1 3SR, UK

**Keywords:** *Drosophila*, behaviour, cellular resolution, optogenetics, thermogenetics, reporters

## Abstract

The fruit fly *Drosophila melanogaster* has emerged as a popular model to investigate fundamental principles of neural circuit operation. The sophisticated genetics and small brain permit a cellular resolution understanding of innate and learned behavioural processes. Relatively recent genetic and technical advances provide the means to specifically and reproducibly manipulate the function of many fly neurons with temporal resolution. The same cellular precision can also be exploited to express genetically encoded reporters of neural activity and cell-signalling pathways. Combining these approaches in living behaving animals has great potential to generate a holistic view of behavioural control that transcends the usual molecular, cellular and systems boundaries. In this review, we discuss these approaches with particular emphasis on the pioneering studies and those involving learning and memory.

## 1. Introduction

The appreciation that behaviours are orchestrated by functioning neural circuits has led to several large-scale projects that are attempting to map neural diagrams of mammalian and insect brains [1–4]. Although these static views of circuit architecture, or connectomes, are important road maps, they will not explain behavioural control. Even the relatively simple 302 neuron connectome of the round worm *Caenorhabditis elegans*, which has been known for 30 years [5,6], is insufficient to explain the animal's behaviour because internal states and experience modulate and alter the efficacy of the neural networks [7]. Therefore, one needs to understand functional connectivity—how individual neurons and neuronal assemblies operate together—within the brain.

This is not a trivial task by any means. On top of a connectome, one needs to assign the mode of signalling to each component neuron, have an appreciation of the strength of connections between neurons, in addition to learning how different behavioural states of the animal alter the neural circuit dynamics. It perhaps seems obvious that achieving such a complete picture of a brain is easier when studying animals, such as invertebrates, that have a relatively small nervous system. These numerically reduced systems are likely to provide the first opportunities to model realistic brain function and to understand how adaptive and context-appropriate behavioural control arises. A small number of neurons is not the only desirable feature, because deciphering neural circuit function requires intervention. Recent revolutionary developments allow investigators to switch identified neurons on and off while recording consequences in larger neural networks, as the animal behaves. Many of these tools and approaches were first demonstrated in research using the fruit fly *Drosophila* as a model, and these will be emphasized in this review.

At the heart of all of the new developments has been the general concept that biology can be understood by harnessing the numerous intricate cell biological processes that have arisen across species; shaped and honed by the selective pressures of evolution. In this review, we will discuss how some of these highly evolved mechanisms from a multitude of organisms, including the fly itself, have been exploited as transgenic tools. By expressing them in either a heterologous or ectopic manner, fly researchers have probed how neural circuit activity translates to behavioural control, and even how memory is used.

## 2. The fruit fly as a model for behaviour

Behaviour has been studied in the fruit fly since the pioneering neurogenetic studies in the early 1970s by the late Seymour Benzer and colleagues [8]. The principle was straightforward—the same mutagenesis strategies that uncovered mysteries of developmental biology [9,10] would yield insight into the generation of behaviour. These early efforts in the Benzer laboratory initiated the field by jumping into some of the most interesting areas, such as circadian rhythms [11], courtship [12] and learning and memory [13]. More recently, studies have extended to include feeding [14–16], aggression [17], sleep [18,19] and motivation [20,21], as well as longevity [22] and neurodegenerative conditions [23–25].

**Figure 1.**
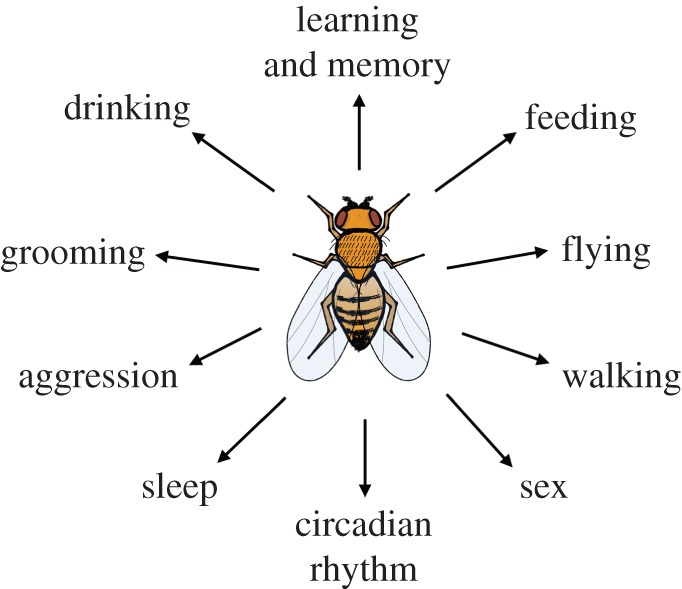
Schematic illustrating some of the many behaviours that have been investigated using fruit fly genetics. Flies must decide which of the homeostatic behaviours, sleep, feed, drink, mate, fight and groom, to preferentially engage in and which mode of locomotion, walk, jump or fly, to employ to accomplish getting where they need to go. They can also adjust their strategy through learning.

We now know that in addition to being a fantastic genetic model, the fruit fly nervous system has an intermediate numerical complexity to the worm or mouse, making it an appropriate model to study conserved neural circuit underpinnings controlling a fairly sophisticated behavioural repertoire. The approximately 100 000 neurons of the fly brain orchestrate behaviours that facilitate the survival and propagation of the species (figure 1). Recent genetic tools now allow one to reproducibly and specifically manipulate the activity of many neuron types in the fly brain. This ability to directly influence the function of specific cells is a key feature of the studies emphasized here.

## 3. Cell-specific gene expression

**Figure 2.**
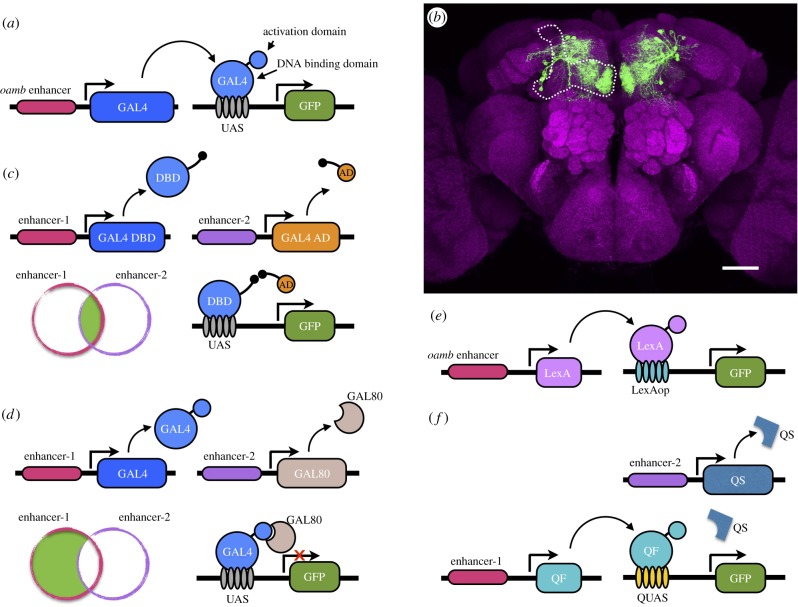
The GAL4, split-GAL4, LexA and QF binary expression systems. (*a*) The GAL4 coding region is either cloned downstream of a promoter stretch (e.g. as shown, from the *oamb* gene), or inserted randomly in the genome on a transposable element. The specificity of the local enhancer confers similar cell-specific expression on GAL4. This source of GAL4 can then be combined with a UAS-driven green fluorescent protein (GFP) transgene to visualize the resulting expression pattern. (*b*) A confocal microscope projection through such a GAL4 line that specifically expresses GFP in a subset of rewarding dopaminergic neurons (green). Additional expression has been removed for illustrative purposes. The brain is generally labelled with an antibody against the synaptic protein Bruchpilot (magenta). Scale bar, 40 μm. (*c*) The specificity of expression can be improved using the split-GAL4 positive intersectional approach where the DNA-binding (DBD) and transactivation domains (AD) are expressed using different enhancers and a functional GAL4 is only reconstituted in cells that express both parts. (*d*) Negative intersection with GAL80 can be used to inhibit GAL4 activity. This control can be constitutive or temperature regulated using GAL80^ts^. (*e*) The binary LexA/LexAop system functions similarly to GAL4 although LexA does not have a regular inhibitory partner. One study intersected its function using RNAi to LexA [21]. Versions also exist where the LexA DBD is fused to the GAL4 or QF AD [26,27]. (*f*) The QF system can be inhibited by the QS protein. Furthermore, QS-mediated inhibition can be temporally regulated by feeding flies with quinic acid.

The first critical step towards controlling cells is to have a means to express effector genes with the desired cellular specificity. Most of these approaches in the fly rely on transposable elements and binary gene expression systems (figure 2). Promoter regions confer cell-type-specific expression to genes that lie downstream. These promoters, and their cell-type-specific expression, can be captured if a transposable *P*-element carrying a reporter gene reading frame inserts downstream [28–30]. The reporter carried by the transposon then ‘enhancer-traps' the promoter and gains expression in the cells that usually express the trapped gene. *P*-elements have been genetically engineered to a fine art in the fly and many variants now exist [31,32]. Critically, their mobilization can be controlled at will, and engineered elements are not capable of moving again in an unassisted manner. This has allowed the generation of thousands of stable fly strains with *P*-elements inserted in unique positions in the fly genome, and that by virtue of position can be used to express other genes in specific cells in the animal.

If the *P*-elements encode a transcription factor, this transcription factor can be used in a binary manner to express another gene with the same cell-type specificity as that governed by the enhancer driving the transcription factor. The critical trick is to use a transcription factor from another organism that lacks a cognate factor in flies. The first version of this was developed using the budding yeast GAL4 transcription factor and the upstream activating sequence (UAS_GAL4_) that is bound by GAL4 driving a reporter gene [33] (figure 2). Selecting a suitable transcription factor for this type of application is not trivial. One needs to make sure the same transcription factor binding site is not used by a homologous or unrelated fly transcription factor; e.g. the budding yeast PHO4 shares a site with c-Myc [34] making it less suitable for this purpose. The GAL4 and UAS-reporter parts were imported into the fly on two engineered *P*-elements [33]. When combined, the cell-type-specifically expressed GAL4 drives expression of the reporter with the same cellular specificity, allowing one to visualize the expression (figure 2).

Rather than relying on random transposon insertion, one can logically select promoter regions from genes that are known to be expressed in the cell type of interest, to drive GAL4 with a similar cell-specificity. For example, the *TH* gene encodes tyrosine hydroxylase, an enzyme that is required for the synthesis of dopamine [35]. Consequently, a fragment from the *TH*-promoter [36] directs gene expression in some dopaminergic neurons (although this not always infallible; in fact, the heavily employed *TH*-GAL4 does not label rewarding dopaminergic neurons [37,38]). Similarly, a fragment from the acetylcholine transporter drives gene expression in many cholinergic neurons [39]. These two examples, however, provide expression control confined to most of the cells that use dopamine or acetylcholine but not to defined subsets of this cell type in a particular region of the brain. This is important because it is clear that anatomically discrete neurons that use the same transmitter have unique functions. This is exemplified by fly dopaminergic neurons; those in the central complex regulate arousal [40–42], whereas others innervating discrete zones in the mushroom body lobes convey positive or negative reinforcement [21,37,38,43,44], or provide motivational/state-dependent control [20]. Clearly, it is critical to control individual dopaminergic neurons to understand how each one functions. Although more precise control can sometimes be obtained with the randomness of different *P*-element strains, the GAL4 system also provides some logic to increase cellular specificity.

In yeast deprived of galactose, GAL80 binds to GAL4 and represses its activity [45]. Ectopic co-expression of GAL80 therefore allows one to inhibit GAL4 activity in the fly [46] (figure 2). Partially overlapping GAL4 and GAL80 expression domains limit GAL4 activity to a subset of cells that only express GAL4. Several useful GAL80 strains exist, such as *Cha*-GAL80, that suppresses expression in cholinergic neurons [47], *TH*-GAL80 in dopaminergic neurons [48], *DvGlut*-GAL80 [49] in glutamatergic neurons and *teashirt*-GAL80 [50] that removes most expression in the ventral ganglion and thus confines expression to the brain.

GAL80 has additional strengths. Classic studies in yeast genetics often screened for temperature-sensitive mutations. Such a temperature-sensitive variant of GAL80, when ubiquitously expressed in the fly, permits temporal control of GAL4-directed gene expression [51,52]. GAL80^ts^ exhibits a similar temperature sensitivity in the fly as it does in yeast, inhibiting GAL4 below 25°C and losing suppression above 29°C [52]. Simply elevating ambient temperature alters the body temperature of the flies and releases GAL80^ts^ inhibition of GAL4. GAL80^ts^ is particularly useful to control the expression of activity-regulating tools that lack intrinsic temporal features, e.g. when ectopically expressing potassium channels such as Kir2.1 [53], EKO [54] or DORK [55] to silence, or at least reduce neural activity, inhibitors or toxins such as tetanus to inhibit exocytosis [56], or pertussis to inhibit G_i_ signalling [57], genetically encoded RNA interference [58,59], or constitutively active dominant negative transgenes.

The general principles of the GAL4-UAS system have been replicated in additional binary systems based on the bacterial LexA transcription factor and its LexAop [26] and the *Neurospora crassa* QF and its QUAS [27,60] (figure 2). In addition, methods exist that allow one to temporally control each of these systems. The QF factor can be suppressed by expression of the *Neurospora* protein QS and the QS inhibition can in turn be relieved by feeding flies with quinic acid [27,60]. A fusion of the LexA DNA-binding domain with either the GAL4 or QF activation domain to form LexA-GAL4AD or LexAQF permits control using the GAL80 or QS systems, whereas a LexA::VP16 version that uses the herpes viral VMW65 activation domain is resistant to GAL80 and QS control [26,27]. Combining the three binary systems in parallel in the same fly allows simultaneous and independent labelling of three sets of neurons, or when combined with the numerous effector and reporter transgenes, described later, permits an amazing combination of parallel and independent circuit manipulations in the same behaving fly.

One can also intersect the binary expression systems in logical ways to limit expression to either cells that are common to the two lines, or cells that are unique to one of the two lines. There are many ways to do this, for example one can use LexA to drive expression of the yeast FLP recombinase which removes a FLP-recombinase target (FRT) sequence flanked transcriptional stop cassette from a UAS_GAL4_ target transgene [61,62]. Therefore, only where LexA overlaps with the GAL4 will the GAL4 be able to drive the UAS_GAL4_ target transgene. Alternatively, the FRT sequences can flank the target transgene reading frame [63] so that the target is not present in the LexA/FLP positive cells and so is only driven in the cells that are unique to the GAL4 line.

A similar intersectional logic can be used with components of a split-GAL4 system. Independent expression of separate DNA-binding and activation domains in partially overlapping patterns only reconstitutes the active transcription factor in the cells that are common [64] (figure 2). Sometimes the resulting cellular specificity is spectacular. A large library of mushroom body-related split-GAL4 lines has recently been published [65]. A split LexA system has also been reported [66] and approaches have been generated that permit exchange of cassettes encoding GAL4, LexA, QF and the split systems within transposable element backbones in a known genomic context [67,68]. This incredible genetic flexibility greatly facilitates production of a customized tool-kit to direct cell-specific expression of effector transgenes.

## 4. Temporal control of specific neurons

**Figure 3.**
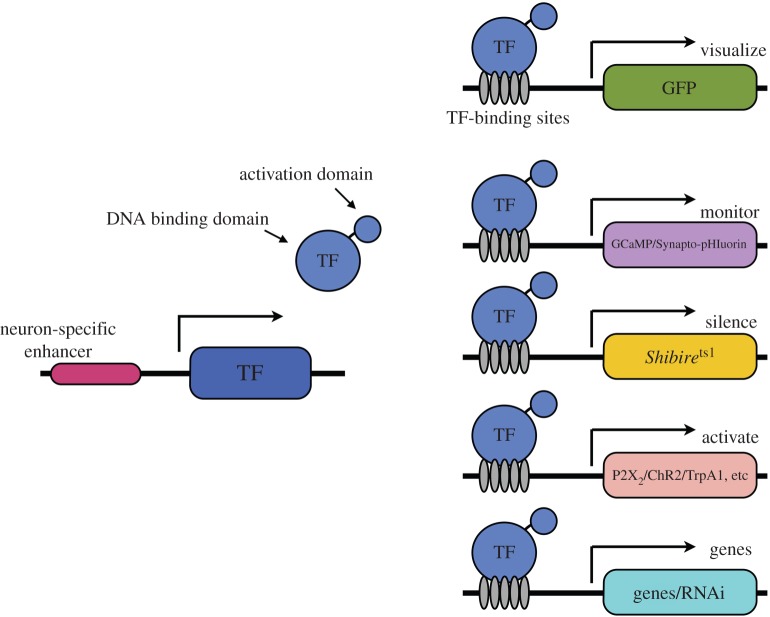
Effector and Reporter transgenes. The GAL4, LexA and QF transcription factors (TF) can be used to express any of a number of functional transgenes that permit visualization, recording, silencing, activation and interference with specific genes.

**Figure 4.**
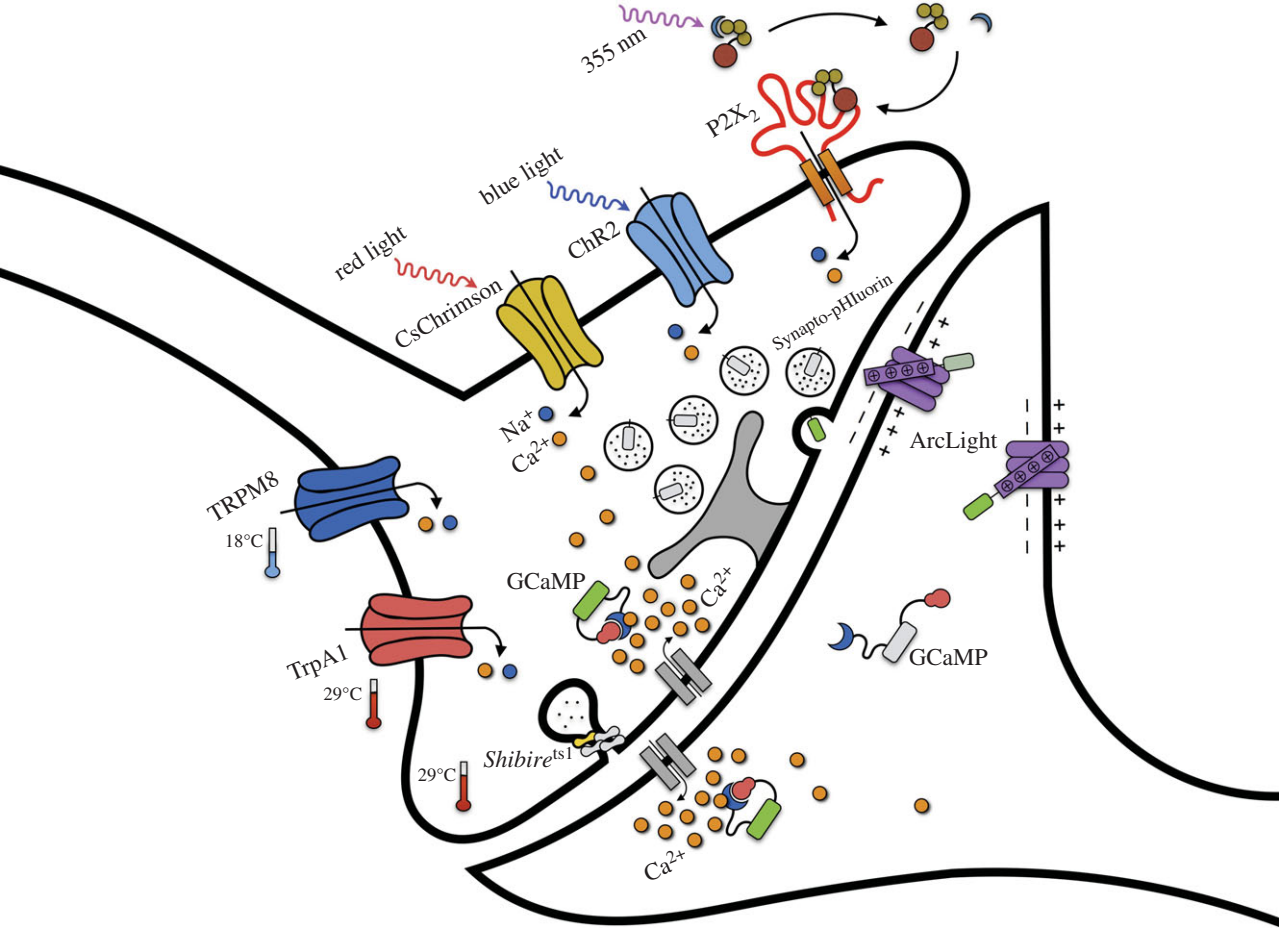
Schematic of a synapse illustrating the main effector and reporter transgenes discussed in the review. The temperature-controlled TRPM8 and dTrpA1, the light-activated CsChrimson and ReaChR (not shown) and the ATP receptor P2X_2_ gate cation influx. The *Shibire*^ts1^ encoded temperature-sensitive dynamin (yellow) is a critical part of the synaptic vesicle exo/endocytosis machinery. The oligomeric nature of dynamin presumably accounts for *Shibire*^ts1^ dominant negativity. Synapto-pHluorin is localized to the synaptic vesicle lumen and its fluorescence increases as the acidified vesicle docks with the plasma membrane and releases the vesicle contents. GCaMP and ArcLight can report neural activation in both the pre- and post-synaptic compartment.

The most successful tools that have been developed can be driven by the GAL4-, LexA- or QF-based systems and provide the ability to control specific neurons with temporal resolution (figure 3). In this section, we will overview the various effectors (figure 4)—putting their development in a historical context. However, the reader should note that beyond discussing the founding examples, other studies have been selected to highlight a particular use, and it is not our objective to provide a comprehensive list here.

### (a) *Shibire*^ts1^-temporal blockade of neurotransmission

*Shibire* was the first genetically encoded tool that permitted temporal control of neural function and it fundamentally altered the way neural circuit analysis is approached in the fly. In a seminal study in the early 1970s, a set of non-complementing temperature-sensitive *Shibire* alleles were discovered [69,70]. Mutant animals showed impaired locomotion at temperatures above 29°C [69]. They also lost the on and off transients in electroretinograms suggesting a possible defect in neural signalling [70]. Importantly, the disruption was reversible; the flies regained wild-type behaviour and physiology within seconds/minutes of being returned to room temperature. When cloned, *Shibire* was shown to interact with the membrane dynamics of endocytosis [71–73] and to encode a dynamin protein [74,75] (figure 4). In addition, at restrictive temperatures, *shi*^ts1^ dynamin has been shown to lead to rapid synaptic fatigue within 20 ms of repetitive stimulation at the dorsal longitudinal muscle neuromuscular synapses of adult *Drosophila* [76]. The groundbreaking development involved cloning the *Shi*^ts1^ coding region under UAS_GAL4_ control [77]. This UAS-*Shi*^ts1^ transgene allowed one to misexpress *Shi*^ts1^ in neurons of choice and transiently block their neurotransmission by elevating the temperature of the flies above the restrictive 29°C [77].

The utility of UAS-*Shi*^ts1^ was first demonstrated in a memory study testing for an acute role of dorsal paired medial (DPM) neurons in memory consolidation [78] and was quickly followed by studies of the mushroom body neurons [79,80]. DPM neurons were identified as expressing the *amnesiac* locus and they project complex processes throughout the mushroom body lobes [78]. This DPM study exemplified a key strength of the temporal control provided by UAS-*Shi*^ts1^. Efforts to ablate the DPM neurons using cell death genes were ineffective because earlier larval expression of the GAL4 line that labels DPM neurons in the adult fly lead to lethality. Instead, acutely blocking DPM neurons in the adult fly using UAS-*Shi*^ts1^ disrupted the stability of olfactory memory; a near phenocopy of the defect observed in *amnesiac* mutant flies [78].

Another invaluable feature of UAS-*Shi*^ts1^ is reversibility. In most cases, normal neural function resumes when the flies are returned from the restrictive to permissive temperature. This was exploited in mushroom body studies that used UAS-*Shi*^ts1^ to temporally limit inactivation to discrete periods of the learning and memory process [79,80]. By only elevating the temperature during either the training, testing or intervening periods of the experiment, mushroom body neurons could be selectively crippled during the memory acquisition, retrieval or consolidation phases. Follow up studies on the mushroom body have led to the general idea that olfactory memory is processed by the anatomically discrete *γ*, *α*′*β*′ and *αβ* subsets of the overall 2000 mushroom body neurons functioning together as a time-sensitive system. This interaction is most clear following appetitive conditioning where neurotransmission from the *γ* neurons is required for short-term memory retrieval, *α*′*β*′ neuron output is required after training with the DPM neurons to stabilize memory, and the *αβ* neurons are most critical for long-term memory retrieval [81–85].

UAS-*Shi*^ts1^ of course has caveats. It is not clear whether it uniformly blocks the release of all synaptic vesicles, such as those that are ‘dense core’ containing monoamine and neuropeptide transmitters. Nevertheless, there are numerous examples where the tool has created a phenotype in dopaminergic and peptidergic neurons. For example, UAS-*Shi*^ts1^ has been instrumental in determining the nature of dopaminergic reinforcement in the fly and it is noteworthy that the mushroom body *αβ* and *γ* neurons express short Neuropeptide F [86], although some phenotypes might result from disruption of the co-release of a currently mysterious fast-transmitter. Even after 15 years, UAS-*Shi*^ts1^ remains the tool of choice to test the importance of specific neurons in a particular neural process.

### (b) Optogenetic neural stimulation

Neuroscience across species has recently been revolutionized by light-triggered neural activation, or as it was later termed ‘optogenetics'. The proof of concept of optogenetics was demonstrated using heterologous expression of components of the fly visual system—arrestin 2, a rhodopsin, and the alpha subunit of a G-protein—in hippocampal neurons [87]. Broad illumination of a population of neurons elicited action potentials only in those cells expressing the ‘chARGe’ system. This study firmly established the precedent that if such switches could be selectively expressed in neurons of interest, they could be specifically activated by light that was generally applied across the brain.

The second pioneering step demonstrated the utility of optogenetics in live-behaving fruit flies. Rather than use the three visual system components of chARGe, the authors expressed the rat ATP-responsive P2X_2_ receptor [88] (figure 4) that does not have a clear equivalent in the fly genome. Neurons expressing P2X_2_ could then be selectively activated in intact flies by photo-release of an injected caged ATP; in effect producing light-controllable animals. The first results were spectacular. Expression of UAS-P2X_2_ in the fly giant fibre neurons led to light evoked take-off, even in flies that lacked a head! In addition, activating the *TH*-GAL4 labelled population of dopaminergic neurons lead to alteration of locomotor behaviour. Another study from the same group used UAS-P2X_2_ to generate male-specific courtship song by activating *fruitless*-expressing neurons in the thoracic ganglion of female flies. Although these females sung ‘out-of-tune’, their song could be perfected if the females also expressed the male-specific *fru*M isoform [50]. These results suggest that a song-generating motor programme exists in female flies, but that it lacks the male physiology and neural commands for song initiation. Another impressive demonstration formed phantom aversive memories by pairing odour exposure with P2X_2_-mediated light-activation of *TH*-GAL4 dopaminergic neurons [43].

The optogenetic tools that are now the most widely used are based on microbial opsins that are integral to cation channels [89,90]. As the name suggests, the Channelrhodopsins (figure 4) provide an easier tool for neuronal activation because the product of a single transgene can be directly gated by light. There is no requirement for photo-uncagable compounds but in many cases the critical all-*trans*-retinal cofactor needs to be provided in the fly's diet. However, until recently, low channel conductance and poor penetration of the fly cuticle of the short wavelength light required to activate Channelrhodopsin have impeded its application to adult fly behavioural studies.

The first optogenetic study of learning in *Drosophila* bypassed the issue of the adult cuticle by expressing a UAS-Channelrhodopsin 2 (ChR2) in transparent larvae [91]. One hundred millisecond light pulses delivered to motor neurons expressing ChR2 evoked activity in body wall muscles at the neuromuscular junction. The authors also expressed ChR2 in either *TH*-GAL4 labelled dopaminergic or *Tdc2*-GAL4 labelled octopaminergic neurons and paired their photoactivation with odour exposure. Whereas dopaminergic neuron activation formed aversive memory, the octopaminergic neuron encoded experience was appetitive [91]. UAS-*ChR2* was also employed to study the consequences of peripheral sensory neuron activation in adult flies [92–94] and it has been successfully used in physiological studies where part of the head cuticle is removed and the brain is directly illuminated [95–98].

Recent technical improvements have made Channelrhodopsin variants that are much more useful for adult fly behaviour. For instance, shifting the activation into the red spectrum in UAS-*ReaChR* [99] increased penetration of the fly cuticle as well as using wavelengths of light that apparently do not interfere with normal fly vision. UAS-*ReaChR* permitted time-resolved activation of courtship song [100]. ReaChR was also employed in a recent study to demonstrate that activating output neurons from the tips of the horizontal lobes of the mushroom body, drives avoidance behaviour [101]. Further novel variants of Channelrhodopsins with distinct properties have been identified by de novo sequencing more than 100 algal transcriptomes [102]. The activation wavelengths of CsChrimson lie within the red spectrum (figure 4). The authors demonstrated CsChrimson's value for studying fly behaviour, by expressing it in projection neurons that mediate CO_2_ avoidance and observing that flies avoid illuminated quadrants of a plate [102]. CsChrimson was also instrumental in a recent study of mushroom body output neurons. CsChrimson-mediated activation of individual sets of mushroom body output neurons was shown to either drive avoidance or approach behaviour [103]. Lastly, mutagenesis has been used to generate ChR2-XXL, a Channelrhodopsin with high expression level and a long open-state [104] and chemical engineering has provided artificial retinal analogues that can alter colour tuning and light sensitivity of ChR2 variants [105].

### (c) Temperature-triggered neural activation

Changes in ambient temperature such as those that are appropriate for inactivating neurotransmission with *Shibire*^ts1^ [77] can also be used to activate neurons that misexpress temperature-sensitive transient receptor potential (TRP) channels. Both the fly heat-activated dTrpA1 [106] and cold-activated rat TRPM8 [107,108] channels have been effectively used in the fly (figure 4).

The fly *dTrpA1* gene encodes a non-specific cation channel that is required in a small number of neurons in the brain for temperature preference [106]. Ectopically expressed UAS-*dTrpA1* depolarizes neurons when flies are exposed to more than 25°C, allowing one to stimulate specific neurons by raising the temperature of the flies. An early study expressed UAS-*dTrpA1* in circadian neurons with *pdf*-GAL4 and found that continuously stimulating these neurons, by housing the flies at 27°C, promoted wakefulness in the early night [109]. More acute activation protocols were used in another study that identified three layers of a neural circuit that provides hunger control of the behavioural expression of sugar-reward memory [20]. UAS-*dTrpA1* mediated activation of Neuropeptide F (dNPF), the fly orthologue of mammalian NPY, producing neurons prior to memory testing mimicked food-deprivation and led to expression of sugar memory even in food satiated flies. By contrast, similarly timed dTrpA1-mediated activation of the mushroom body innervating dopaminergic MB-MP1 neurons suppressed sugar memory retrieval in hungry flies.

In another striking study, dTrpA1-mediated activation of dorsal fan-shaped body neurons was shown to be sufficient to put flies to sleep. Furthermore, the artificially induced sleep was capable of facilitating long-term memory formation [110].

Acute neural activation with UAS-*dTrpA1* has also been instrumental in studies of reinforcement signalling during learning. As briefly mentioned above, blocking dopaminergic neurons with UAS-*Shi*^ts1^ implicated specific groups of these neurons in aversive [111] and rewarding reinforcement [37,38] during olfactory conditioning. Furthermore, the optogenetic study of Claridge-Chang *et al.* [43] revealed that dopaminergic neurons in the PPL1 cluster were those that were likely to be sufficient to provide aversive reinforcement. Further studies with dTrpA1-mediated activation established that the MP1 and MV1 neurons within PPL1 have reinforcing properties [44], in addition to the MB-M3 neurons from an anatomically discrete cluster called PAM [112]. dTrpA1-mediated neural activation combined with UAS-*Shi*^ts1^ experiments also facilitated the discovery of rewarding dopaminergic neurons in the PAM cluster. Pairing their heat-activation with odour formed robust odour approach behaviour, whereas blocking them compromised sugar- or water-rewarded learning [21,37,38,113,114].

To date, the cold and menthol-activated TRPM8 has been less frequently employed [107,108,115]. In the original fly study, UAS-*TRPM8* was driven in neurons that express the CCAP neuropeptide and activation of CCAP neurons by placing the flies at 15°C was shown to induce wing expansion in newly eclosed adults [116]. UAS-*TRPM8* was also used to corroborate the dTrpA1 findings that activation of the MB-MP1 dopaminergic neurons suppressed appetitive memory expression in hungry flies [20].

Ectopic expression of UAS-*TRPM8* or UAS-*dTrpA1* driven by large GAL4 collections has also been used to screen for neurons contributing to a wide-range of behaviours, such as feeding, walking, grooming, courtship, copulation and aggression [117–124]. Lastly, a clever recent study showed that dTrpA1-mediated activation of random collections of mushroom body Kenyon cells could be paired with an electric shock punishment to induce aversive memories; with the flies subsequently avoiding the zone of a temperature gradient that would lead to the reactivation of these same neurons [125].

In principle, it should be possible to combine dTrpA1 and TRPM8 tools by using the different binary expression systems, to express them in discrete sets of neurons of the same fly. These neurons could then be independently controlled with the relevant changes in temperature required to activate the two TRP channels.

It is important to note that the onset of activity is much slower with heat than it is with light control, and that opto- and thermogenetic stimulation will not always provide similar results. For instance, a separation between a deterministic and a probabilistic component of male courtship song was evident using UAS-*ReaChR* mediated activation of neurons but not using thermogenetic UAS-*dTrpA1* [100]. Most of the optogenetic and thermogenetic neural stimulation studies discussed above stimulate particular neurons without detailed consideration of the firing dynamic. The obvious success of these studies therefore surprisingly questions the importance of temporal activity patterns in these neurons. It will be interesting to record from neurons during stimulation with these tools and to compare the evoked activity patterns with those generated by physiologically relevant stimuli. It is conceivable that some neurons are constrained to adopt one of a few possible firing states, regardless of the activity that is injected with the optogenetic or thermogenetic actuators. Another potential issue of artificially induced firing is the observation that excessive firing can put neurons into a refractory depolarization block period where they do not fire at all [126]. In such a case, investigators might be misled to think an observed phenotype results from excitation when in fact the neurons have been inhibited.

## 5. Recording circuit physiology

Stimulation and inhibition techniques allow one to assemble a low-resolution idea of how certain neural circuits are ordered and operate to direct behaviour. These models can be challenged and extended by using the same cell-specific expression control to produce a number of genetically encoded reporters of neural activity and cell-signalling processes (figures 3 and 4).

Despite having lower temporal resolution, genetically encoded reporters do have advantages over single electrode electrophysiology. They can be relatively easily and reproducibly targeted to the same cell type and with the right control, they can facilitate recording from specific cells or neural ensembles in the small fly brain. Lastly, they can be monitored by a somewhat less invasive procedure that only requires a small window to be opened in the head cuticle of a fly that is mounted in a suitable orientation for viewing under the microscope and that permits the fly to respond to the relevant stimuli. All of the currently popular reporters rely on variants of fluorescent proteins that ‘report’ activity, or a particular cellular event, by changing their emission. The difficulties in their use are therefore mostly related to recording fluorescence. Although the animal is mounted under the optics of a suitable microscope, movement of the tissue needs to be minimized and accounted for because subtle shifts of the focal plane lead to measurable changes in fluorescence. This is a particular issue if the sensitivity and signal-to-noise ratio of the reporter is low. One way to account for that is to co-image a second non-activity reporting fluorescent marker in the same focal plane. Lastly, as some of the reporters bind ions or metabolites, they could potentially buffer, and therefore disrupt, the cellular process that they are designed to record. Nevertheless, the advantages of genetically encoded reporters largely outweigh the concerns. The ability to specifically express the reporter of choice in the neurons of interest means that targeted recording is readily and reproducibly achievable. At present, the most commonly used reporters monitor changes in calcium concentration [127–133], synaptic vesicle fusion [134], second messengers (such as cAMP [135]) or voltage [136,137] (figure 4). Most of these tools have been used to observe spontaneous or stimulus-evoked responses, as well as to measure neural responses to artificial stimulation of potentially afferent neurons.

A pioneering study used the GAL4-UAS system to express the calcium-sensitive luminescent protein apoaequorin in *Drosophila* Kenyon cells [138]. Surprisingly, when the essential cofactor coelenterazine was added to dissected brains in a luminometer, the mushroom bodies exhibited a synchronous oscillation in intracellular calcium; a phenomenon that was altered in brains taken from forgetful *amnesiac* mutant flies. Three landmark studies imaged odour-evoked neural activity in the antennal lobe using either live fly or partly dissected fly head preparations. Expression of the ratiometric calcium sensor UAS-cameleon 2.1 [127] in projection neurons measured odour-evoked activity in the antennal lobes and mushroom body calyces [139]. A more detailed study [140] measured synaptic transmission at multiple layers of the olfactory system by expressing the synaptic vesicle localized pH-sensitive UAS-Synapto-pHluorin (spH) in olfactory sensory neurons, projection neurons and inhibitory local neurons in the antennal lobes. Natural odours were seen to elicit similar combinatorial patterns of activity in the sensory neuron and projection neuron layers of the antennal lobe suggesting a faithful transmission of odour information within identifiable glomeruli [140]. In addition, the ability to record synaptic transmission revealed that projection neurons have recurrent synapses in the antennal lobe and that local neurons provide broad interglomerular inhibition. A similar conserved sensory-projection neuron activation was also observed using the calcium-sensitive UAS-GCaMP reporter [141].

Although Synapto-pHluorin provides a more direct measure of vesicle dynamics than presynaptic calcium influx, the increased sensitivity and improved speed of a new generation of GCaMP reporters (GCaMP6 can detect single action potentials) [133] has made GCaMP the most popular activity reporter. However, GCaMP and spH have both been heavily employed at all levels of the neural circuitry involved in olfactory memory. For example, several studies have identified physiological changes in odour-evoked responses after training, or ‘memory traces' in the mushroom body neurons [142–145], and the mushroom body-associated DPM and anterior paired lateral (APL) neurons [146–148], some of which are altered in memory defective flies [146,149], suggesting their plausible importance in learned behaviour. Other applications have included the visualization of sparse odour coding in the Kenyon cell population [150–152], reward and punishment signalling in reinforcing dopaminergic neurons [21,37,38,153,154], activity in dopaminergic neurons after training [153,155,156] and learning-induced changes in odour-evoked drive to neurons downstream of the mushroom body [101,157–159].

As calcium is only ever a surrogate for neural activity, and direct physiological recording is tricky for single cells and impossible for larger ensembles, there has been great interest in developing genetically encoded fast voltage sensors (figure 4). There is still someway to go, but the recently described ArcLight and FRET-opsin reporters suggest robust millisecond resolution voltage recording will be achievable [136,137,160]. The ideal voltage sensor would partially replace the need for classical electrophysiology and make it possible to perform multi-channel ensemble recording in the small fly brain.

Genetically encoded sensors can also allow one to monitor changes in cAMP second messenger signalling. These are particularly useful in neuroscience because several neuropeptide, monoamine and fast-transmitters evoke metabotropic responses in recipient neurons through G-protein-coupled receptors. The Epac1-camps cAMP sensor [135] was first employed in fly circadian neurons to show a modulatory effect of the PDF neuropeptide [161] and to later determine that the clock network comprises multiple independent oscillators [162]. Epac1-camps and a Protein kinase A sensor AKAR2 [163] have also been used to measure the effect of extraneous dopamine on mushroom body neurons [164,165]. Finally, genetic approaches also exist to mark cells that are subject to dopaminergic modulation [166].

## 6. Combining the approaches to assemble functional neural pathways

Opto- and thermogenetic stimulation and inhibition and recording studies permit the identification of component neurons that contribute to behaviours but do not themselves provide information about neural circuitry. It is necessary to understand how they are embedded within a larger context—what neurons lie upstream and downstream. Knowing the neurotransmitter a particular neuron uses can be very helpful for this purpose. If neural stimulation allows one to generate an overt behavioural change, such as mimicking a change in the animals state or forming a memory, one can assume that the relevant downstream neurons must express receptors to receive the signals. Cell-specific expression of RNA interference constructs to neurotransmitter receptors can therefore be used to identify the sites in the brain where the gain of function transmitters act, and therefore to map functional connectivity. This is more difficult for fast-transmitters because of the complexity of the number and subunit composition of their receptors but it is fairly straightforward for neuropeptides and monoamines, which often exert their function via single or small numbers of receptors that are single subunits.

As previously discussed, dTrpA1-mediated activation of peptidergic dNPF neurons conferred a food-deprived like state that promoted the behavioural expression of appetitive memory [20]. After finding that loss of the dNPF receptor *npfr1* gene locked the flies in an apparently food-satiated state and inhibited appetitive memory expression, the authors used cell-type-restricted expression of UAS-*npfr1*^RNAi^ to identify where dNPF acts. They identified the MB-MP1 dopaminergic neurons as a key site where dNPF signalling is required to gate appetitive memory expression. As noted earlier, UAS-*Shi*^ts1^ and UAS-*dTrpA1*/*TRPM8* experiments subsequently established an inhibitory mode of operation for MB-MP1 neurons, thus leading the authors to propose a hierarchical inhibition circuit motif [20]. Hunger promotes the release of dNPF, which releases the inhibitory influence of MB-MP1 dopaminergic neurons to facilitate the expression of food-relevant memories.

If stimulation of a particular set of neurons results in an ectopic behaviour, the relevant downstream receptor can also be identified, by testing whether the activation phenotype remains in a receptor mutant background. For example, pairing odour exposure with UAS-*dTrpA1* mediated activation of *Tdc2*-GAL4 labelled octopaminergic neurons formed a labile appetitive memory, but it could not be formed in flies that were compromised for either the *Oamb* alpha-adrenergic-like receptor or the *oct*β*2R* beta-adrenergic-like receptor [38]. These two receptors appear to exert their learning-related functions in different subsets of reinforcing dopaminergic neurons. Consequently, *Tdc2*-GAL4 activation also failed to implant reward memory in a *dumb*^1^ mutant background that lacks the D1-like dopamine receptor.

A similar mutant background approach established that the aggression-promoting effects of dTrpA1-mediated activation of Tachykinin-releasing neurons required the function of the *Takr86C*-encoded receptor [124].

With sufficient and independent cell-specific control, investigators can combine as many of the described tools as they can muster in one fly brain. For example, calcium imaging can be combined with UAS-*dTrpA1* and UAS-*Shi*^ts1^-mediated activation or inactivation of neural subtypes to understand functional connectivity. Such an approach was used in a recent study that uncovered feedback inhibition within the mushroom bodies [152]. Odour-evoked activity was higher at the restrictive temperature in mushroom body neurons that coexpressed UAS-*GCaMP3* and UAS-*Shi*^ts1^. The feedback comes from the GABA-ergic APL neurons that have elaborate processes throughout the mushroom body. Expressing lexAop-*dTrpA1* in Kenyon cells and UAS-*GCaMP3* or UAS-*spH* in APL neurons revealed that the mushroom body drives the APL neurons. Further experiments that required intersectional genetics to express UAS-*Shi*^ts1^ or UAS-*dTrpA1* cleanly in APL neurons and lexAop-*GCAMP3* in Kenyon cells revealed that APL neurons inhibit odour-evoked activity in the mushroom body, thereby closing the feedback loop.

A similar strategy was used to investigate the effects of dopamine release onto the mushroom body [167]. Discrete subsets of dopaminergic neurons were activated using UAS-*dTrpA1* expressed under the control of various GAL4 drivers. Cyclic AMP levels or PKA activity were simultaneously monitored in the mushroom body neurons expressing either Epac1-camps [135], ^T^Epac^VV^ [168] or the PKA-reporter AKAR3 [169], respectively. Alternatively the Ca^2+^ sensor GCaMP3 driven by a mushroom body neuron-restricted promoter was used to measure odour-evoked activity following an artificial learning paradigm pairing odour-presentation and dTrpA1-mediated activation of dopaminergic neurons [167]. The use of temperature-regulated tools in combination with imaging is thus a powerful approach, although temperature-induced movement can make the imaging of small processes tricky.

An alternative to thermogenetics is to combine optogenetics or chemogenetics with imaging or electrophysiology. For example, application of ATP onto mushroom body neurons that express UAS-P2X_2_ while recording electrophysiologically from projection neurons and local neurons in the antennal lobe suggests a functional feedback within these layers of the olfactory circuit in the fly [170]. Using a similar approach, glutamate was shown to be an inhibitory neurotransmitter in the fly antennal lobe. Activating glutamatergic local neurons using UAS-P2X_2_ expression and ATP application while recording from projection neurons revealed a hyperpolarizing response [171]. UAS/lexAop-P2X_2_-mediated activation of *pdf*-GAL4 expressing circadian neurons was also successfully employed in combination with GCaMP and Epac1-camps imaging in putative follower neurons in the clock system [162].

Channelrhodopsins have also been useful in such endeavours. An impressive study combined GCaMP imaging of individual dendritic claws on a single Kenyon cell with UAS-ChR2 optogenetic activation of subsets of projection neurons and electrophysiological recordings of Kenyon cells [95]. The authors found that each of the average of seven claws per Kenyon cell responded as an individual, and that activity in three to four of them was likely to drive the cell to spike.

Another study demonstrated functional connectivity in the motion detection part of the fly visual system. UAS-ChR2 was expressed in small-field T4 and T5 cells and electrophysiological recordings were made from lobula plate tangential cells [172].

## 7. Closing remarks

Beyond being a fabulous test-bed for new genetically encoded tools, the future looks very bright for studies of neural circuit function in the fly. Many studies already suggest that important neuroscience questions can be addressed and that conserved mechanisms will be revealed that have general relevance.

With improved resolution of microscopy and tools that are localized to cellular subcompartments, one can foresee the ability to combine systems neuroscience and synaptic physiology in the brain. Recent studies in the more accessible preparation of the larval neuromuscular junction have for example used synaptically targeted GCaMP3 to distinguish between spontaneous and evoked modes of neurotransmission [173,174]. Others have employed ChR2 or a light-gated ionotropic glutamate receptor to fine-tune synaptic transmission at the neuromuscular junction [175,176]. In principle then, it should be feasible to combine on and off switches with both detailed synaptic recordings and behavioural analyses. This will not be trivial but tethered fly preparations have already been developed that permit neural manipulation and recording while the animal is still able to fly [177] or walk [178].

## References

[1] Takemura SY 2013 A visual motion detection circuit suggested by *Drosophila* connectomics. Nature 500, 175–181. (10.1038/nature12450) 23925240 PMC3799980

[2] Kleinfeld D 2011 Large-scale automated histology in the pursuit of connectomes. J. Neurosci. 31, 16 125–16 138. (10.1523/JNEUROSCI.4077-11.2011) PMC3758571 22072665

[3] Toga AW Clark KA Thompson PM Shattuck DW Van Horn JD 2012 Mapping the human connectome. Neurosurgery 71, 1–5. (10.1227/NEU.0b013e318258e9ff) 22705717 PMC3555558

[4] Kuan L 2015 Neuroinformatics of the *Allen Mouse Brain Connectivity Atlas*. Methods 73, 4–17. (10.1016/j.ymeth.2014.12.013) 25536338

[5] White JG Southgate E Thomson JN Brenner S 1986 The structure of the nervous system of the nematode *Caenorhabditis elegans*. Phil. Trans. R. Soc. Lond. B 314, 1–340. (10.1098/rstb.1986.0056) 22462104

[6] Varshney LR Chen BL Paniagua E Hall DH Chklovskii DB 2011 Structural properties of the *Caenorhabditis elegans* neuronal network. PLoS Comput. Biol. 7, e1001066 (10.1371/journal.pcbi.1001066) 21304930 PMC3033362

[7] Bargmann CI Marder E 2013 From the connectome to brain function. Nat. Methods 10, 483–490. (10.1038/nmeth.2451) 23866325

[8] Weiner J 2014 Time, love, memory: a great biologist and his quest for the origins of behavior. New York, NY: Vintage Books.

[9] Lewis EB 1978 A gene complex controlling segmentation in *Drosophila*. Nature 276, 565–570. (10.1038/276565a0) 103000

[10] Nusslein-Volhard C Wieschaus E 1980 Mutations affecting segment number and polarity in *Drosophila*. Nature 287, 795–801. (10.1038/287795a0) 6776413

[11] Konopka RJ Benzer S 1971 Clock mutants of *Drosophila melanogaster*. Proc. Natl Acad. Sci. USA 68, 2112–2116. (10.1073/pnas.68.9.2112) 5002428 PMC389363

[12] Hotta Y Benzer S 1976 Courtship in *Drosophila* mosaics: sex-specific foci for sequential action patterns. Proc. Natl Acad. Sci. USA 73, 4154–4158. (10.1073/pnas.73.11.4154) 825859 PMC431365

[13] Quinn WG Harris WA Benzer S 1974 Conditioned behavior in *Drosophila melanogaster*. Proc. Natl Acad. Sci. USA 71, 708–712. (10.1073/pnas.71.3.708) 4207071 PMC388082

[14] Ja WW Carvalho GB Mak EM de la Rosa NN Fang AY Liong JC Brummel T Benzer S 2007 Prandiology of *Drosophila* and the CAFE assay. Proc. Natl Acad. Sci. USA 104, 8253–8256. (10.1073/pnas.0702726104) 17494737 PMC1899109

[15] Al-Anzi B Sapin V Waters C Zinn K Wyman RJ Benzer S 2009 Obesity-blocking neurons in *Drosophila*. Neuron 63, 329–341. (10.1016/j.neuron.2009.07.021) 19679073 PMC2742587

[16] Al-Anzi B Armand E Nagamei P Olszewski M Sapin V Waters C Zinn K Wyman RJ Benzer S 2010 The leucokinin pathway and its neurons regulate meal size in *Drosophila*. Curr. Biol. 20, 969–978. (10.1016/j.cub.2010.04.039) 20493701 PMC2896026

[17] Chen S Lee AY Bowens NM Huber R Kravitz EA 2002 Fighting fruit flies: a model system for the study of aggression. Proc. Natl Acad. Sci. USA 99, 5664–5668. (10.1073/pnas.082102599) 11960020 PMC122828

[18] Shaw PJ Cirelli C Greenspan RJ Tononi G 2000 Correlates of sleep and waking in *Drosophila melanogaster*. Science 287, 1834–1837. (10.1126/science.287.5459.1834) 10710313

[19] Hendricks JC 2000 Rest in *Drosophila* is a sleep-like state. Neuron 25, 129–138. (10.1016/S0896-6273(00)80877-6) 10707978

[20] Krashes MJ DasGupta S Vreede A White B Armstrong JD Waddell S 2009 A neural circuit mechanism integrating motivational state with memory expression in *Drosophila*. Cell 139, 416–427. (10.1016/j.cell.2009.08.035) 19837040 PMC2780032

[21] Lin S Owald D Chandra V Talbot C Huetteroth W Waddell S 2014 Neural correlates of water reward in thirsty *Drosophila*. Nat. Neurosci. 17, 1536–1542. (10.1038/nn.3827) 25262493 PMC4213141

[22] Lin YJ Seroude L Benzer S 1998 Extended life-span and stress resistance in the *Drosophila* mutant *methuselah*. Science 282, 943–946. (10.1126/science.282.5390.943) 9794765

[23] Buchanan RL Benzer S 1993 Defective glia in the *Drosophila* brain degeneration mutant *drop-dead*. Neuron 10, 839–850. (10.1016/0896-6273(93)90200-B) 8494644

[24] Min KT Benzer S 1997 *Spongecake* and *eggroll*: two hereditary diseases in *Drosophila* resemble patterns of human brain degeneration. Curr. Biol. 7, 885–888. (10.1016/S0960-9822(06)00378-2) 9382801

[25] Kretzschmar D Hasan G Sharma S Heisenberg M Benzer S 1997 The swiss cheese mutant causes glial hyperwrapping and brain degeneration in *Drosophila*. J. Neurosci. 17, 7425–7432. 9295388 10.1523/JNEUROSCI.17-19-07425.1997 PMC6573436

[26] Lai SL Lee T 2006 Genetic mosaic with dual binary transcriptional systems in *Drosophila*. Nat. Neurosci. 9, 703–709. (10.1038/nn1681) 16582903

[27] Riabinina O 2015 Improved and expanded Q-system reagents for genetic manipulations. Nat. Methods 12, 219–222. (10.1038/nmeth.3250) 25581800 PMC4344399

[28] O'Kane CJ Gehring WJ 1987 Detection in situ of genomic regulatory elements in *Drosophila*. Proc. Natl Acad. Sci. USA 84, 9123–9127. (10.1073/pnas.84.24.9123) 2827169 PMC299704

[29] Kaiser K 1993 Second generation enhancer traps. Curr. Biol. 3, 560–562. (10.1016/0960-9822(93)90058-V) 15335704

[30] Yang MY Armstrong JD Vilinsky I Strausfeld NJ Kaiser K 1995 Subdivision of the *Drosophila* mushroom bodies by enhancer-trap expression patterns. Neuron 15, 45–54. (10.1016/0896-6273(95)90063-2) 7619529

[31] Rubin GM Spradling AC 1982 Genetic transformation of *Drosophila* with transposable element vectors. Science 218, 348–353. (10.1126/science.6289436) 6289436

[32] Rubin GM Spradling AC 1983 Vectors for P element-mediated gene transfer in *Drosophila*. Nucleic Acids Res. 11, 6341–6351. (10.1093/nar/11.18.6341) 6312420 PMC326377

[33] Brand AH Perrimon N 1993 Targeted gene expression as a means of altering cell fates and generating dominant phenotypes. Development 118, 401–415. 8223268 10.1242/dev.118.2.401

[34] Fisher F Jayaraman PS Goding CR 1991 C-myc and the yeast transcription factor PHO4 share a common CACGTG-binding motif. Oncogene 6, 1099–1104. 1861859

[35] Budnik V White K 1987 Genetic dissection of dopamine and serotonin synthesis in the nervous system of *Drosophila melanogaster*. J. Neurogenet. 4, 309–314. (10.3109/01677068709102351) 3126282

[36] Friggi-Grelin F Coulom H Meller M Gomez D Hirsh J Birman S 2003 Targeted gene expression in *Drosophila* dopaminergic cells using regulatory sequences from tyrosine hydroxylase. J. Neurobiol. 54, 618–627. (10.1002/neu.10185) 12555273

[37] Liu C 2012 A subset of dopamine neurons signals reward for odour memory in *Drosophila*. Nature 488, 512–516. (10.1038/nature11304) 22810589

[38] Burke CJ 2012 Layered reward signalling through octopamine and dopamine in *Drosophila*. Nature 492, 433–437. (10.1038/nature11614) 23103875 PMC3528794

[39] Kitamoto T Ikeda K Salvaterra PM 1995 Regulation of choline acetyltransferase/lacZ fusion gene expression in putative cholinergic neurons of *Drosophila melanogaster*. J. Neurobiol. 28, 70–81. (10.1002/neu.480280107) 8586966

[40] Lebestky T 2009 Two different forms of arousal in *Drosophila* are oppositely regulated by the dopamine D1 receptor ortholog DopR via distinct neural circuits. Neuron 64, 522–536. (10.1016/j.neuron.2009.09.031) 19945394 PMC2908595

[41] Ueno T Tomita J Tanimoto H Endo K Ito K Kume S Kume K 2012 Identification of a dopamine pathway that regulates sleep and arousal in *Drosophila*. Nat. Neurosci. 15, 1516–1523. (10.1038/nn.3238) 23064381

[42] Liu Q Liu S Kodama L Driscoll MR Wu MN 2012 Two dopaminergic neurons signal to the dorsal fan-shaped body to promote wakefulness in *Drosophila*. Curr. Biol. 22, 2114–2123. (10.1016/j.cub.2012.09.008) 23022067 PMC3505250

[43] Claridge-Chang A Roorda RD Vrontou E Sjulson L Li H Hirsh J Miesenböck G 2009 Writing memories with light-addressable reinforcement circuitry. Cell 139, 405–415. (10.1016/j.cell.2009.08.034) 19837039 PMC3920284

[44] Aso Y 2012 Three dopamine pathways induce aversive odor memories with different stability. PLoS Genet. 8, e1002768 (10.1371/journal.pgen.1002768) 22807684 PMC3395599

[45] Nogi Y Shimada H Matsuzaki Y Hashimoto H Fukasawa T 1984 Regulation of expression of the galactose gene cluster in *Saccharomyces cerevisiae*. II. The isolation and dosage effect of the regulatory gene GAL80. Mol. Gen. Genet. 195, 29–34. (10.1007/BF00332719) 6092855

[46] Lee T Luo L 1999 Mosaic analysis with a repressible cell marker for studies of gene function in neuronal morphogenesis. Neuron 22, 451–461. (10.1016/S0896-6273(00)80701-1) 10197526

[47] Kitamoto T 2002 Conditional disruption of synaptic transmission induces male-male courtship behavior in *Drosophila*. Proc. Natl Acad. Sci. USA 99, 13 232–13 237. (10.1073/pnas.202489099) 12239352 PMC130616

[48] Sitaraman D Zars M Laferriere H Chen Y-C Sable-Smith A Kitamoto T Rottinghaus GE Zars T 2008 Serotonin is necessary for place memory in *Drosophila*. Proc. Natl Acad. Sci. USA 105, 5579–5584. (10.1073/pnas.0710168105) 18385379 PMC2291120

[49] Bussell JJ Yapici N Zhang SX Dickson BJ Vosshall LB 2014 Abdominal-B neurons control *Drosophila* virgin female receptivity. Curr. Biol. 24, 1584–1595. (10.1016/j.cub.2014.06.011) 24998527 PMC4476023

[50] Clyne JD Miesenbock G 2008 Sex-specific control and tuning of the pattern generator for courtship song in *Drosophila*. Cell 133, 354–363. (10.1016/j.cell.2008.01.050) 18423205

[51] Waddell S Quinn WG 2001 Flies, genes, and learning. Annu. Rev. Neurosci. 24, 1283–1309. (10.1146/annurev.neuro.24.1.1283) 11520934

[52] McGuire SE Le PT Osborn AJ Matsumoto K Davis RL 2003 Spatiotemporal rescue of memory dysfunction in *Drosophila*. Science 302, 1765–1768. (10.1126/science.1089035) 14657498

[53] Baines RA Uhler JP Thompson A Sweeney ST Bate M 2001 Altered electrical properties in *Drosophila* neurons developing without synaptic transmission. J. Neurosci. 21, 1523–1531. 11222642 10.1523/JNEUROSCI.21-05-01523.2001 PMC6762927

[54] White BH Osterwalder TP Yoon KS Joiner WJ Whim MD Kaczmarek LK Keshishian H 2001 Targeted attenuation of electrical activity in *Drosophila* using a genetically modified K^+^ channel. Neuron 31, 699–711. (10.1016/S0896-6273(01)00415-9) 11567611

[55] Nitabach MN Blau J Holmes TC 2002 Electrical silencing of *Drosophila* pacemaker neurons stops the free-running circadian clock. Cell 109, 485–495. (10.1016/S0092-8674(02)00737-7) 12086605

[56] Sweeney ST Broadie K Keane J Niemann H O'Kane CJ 1995 Targeted expression of tetanus toxin light chain in *Drosophila* specifically eliminates synaptic transmission and causes behavioral defects. Neuron 14, 341–351. (10.1016/0896-6273(95)90290-2) 7857643

[57] Ferris J Ge H Liu L Roman G 2006 G(o) signaling is required for *Drosophila* associative learning. Nat. Neurosci. 9, 1036–1040. (10.1038/nn1738) 16845387

[58] Clemens JC Worby CA Simonson-Leff N Muda M Maehama T Hemmings BA Dixon JE 2000 Use of double-stranded RNA interference in *Drosophila* cell lines to dissect signal transduction pathways. Proc. Natl Acad. Sci. USA 97, 6499–6503. (10.1073/pnas.110149597) 10823906 PMC18635

[59] Dietzl G 2007 A genome-wide transgenic RNAi library for conditional gene inactivation in *Drosophila*. Nature 448, 151–156. (10.1038/nature05954) 17625558

[60] Potter CJ Tasic B Russler EV Liang L Luo L 2010 The Q system: a repressible binary system for transgene expression, lineage tracing, and mosaic analysis. Cell 141, 536–548. (10.1016/j.cell.2010.02.025) 20434990 PMC2883883

[61] Shang Y Griffith LC Rosbash M 2008 Light-arousal and circadian photoreception circuits intersect at the large PDF cells of the *Drosophila* brain. Proc. Natl Acad. Sci. USA 105, 19 587–19 594. (10.1073/pnas.0809577105) PMC2596742 19060186

[62] Perrat PN DasGupta S Wang J Theurkauf W Weng Z Rosbash M Waddell S 2013 Transposition-driven genomic heterogeneity in the *Drosophila* brain. Science 340, 91–95. (10.1126/science.1231965) 23559253 PMC3887341

[63] Stockinger P Kvitsiani D Rotkopf S Tirian L Dickson BJ 2005 Neural circuitry that governs *Drosophila* male courtship behavior. Cell 121, 795–807. (10.1016/j.cell.2005.04.026) 15935765

[64] Luan H Peabody NC Vinson CR White BH 2006 Refined spatial manipulation of neuronal function by combinatorial restriction of transgene expression. Neuron 52, 425–436. (10.1016/j.neuron.2006.08.028) 17088209 PMC1713190

[65] Aso Y 2014 The neuronal architecture of the mushroom body provides a logic for associative learning. Elife 3, e04577 (10.7554/eLife.04577) 25535793 PMC4273437

[66] Ting CY Gu S Guttikonda S Lin TY White BH Lee CH 2011 Focusing transgene expression in *Drosophila* by coupling Gal4 with a novel split-LexA expression system. Genetics 188, 229–233. (10.1534/genetics.110.126193) 21368278 PMC3120155

[67] Gohl DM 2011 A versatile *in vivo* system for directed dissection of gene expression patterns. Nat. Methods 8, 231–237. (10.1038/nmeth.1561) 21473015 PMC3079545

[68] Diao F 2015 Plug-and-play genetic access to *Drosophila* cell types using exchangeable exon cassettes. Cell Rep. 10, 1410–1421. (10.1016/j.celrep.2015.01.059) 25732830 PMC4373654

[69] Poodry CA Hall L Suzuki DT 1973 Developmental properties of *shibire*^ts1^: a pleiotropic mutation affecting larval and adult locomotion and development. Dev. Biol. 32, 373–386. (10.1016/0012-1606(73)90248-0) 4208027

[70] Kelly LE Suzuki DT 1974 The effects of increased temperature on electroretinograms of temperature-sensitive paralysis mutants of *Drosophila melanogaster*. Proc. Natl Acad. Sci. USA 71, 4906–4909. (10.1073/pnas.71.12.4906) 4216025 PMC434008

[71] Ikeda K Ozawa S Hagiwara S 1976 Synaptic transmission reversibly conditioned by single-gene mutation in *Drosophila melanogaster*. Nature 259, 489–491. (10.1038/259489a0) 176591

[72] Koenig JH Saito K Ikeda K 1983 Reversible control of synaptic transmission in a single gene mutant of *Drosophila melanogaster*. J. Cell Biol. 96, 1517–1522. (10.1083/jcb.96.6.1517) 6304107 PMC2112451

[73] Kosaka T Ikeda K 1983 Reversible blockage of membrane retrieval and endocytosis in the garland cell of the temperature-sensitive mutant of *Drosophila melanogaster*, shibirets1. J. Cell Biol. 97, 499–507. (10.1083/jcb.97.2.499) 6411734 PMC2112522

[74] van der Bliek AM Meyerowitz EM 1991 Dynamin-like protein encoded by the *Drosophila* shibire gene associated with vesicular traffic. Nature 351, 411–414. (10.1038/351411a0) 1674590

[75] Chen MS Obar RA Schroeder CC Austin TW Poodry CA Wadsworth SC Vallee RB 1991 Multiple forms of dynamin are encoded by shibire, a *Drosophila* gene involved in endocytosis. Nature 351, 583–586. (10.1038/351583a0) 1828536

[76] Kawasaki F Hazen M Ordway RW 2000 Fast synaptic fatigue in shibire mutants reveals a rapid requirement for dynamin in synaptic vesicle membrane trafficking. Nat. Neurosci. 3, 859–860. (10.1038/78753) 10966613

[77] Kitamoto T 2001 Conditional modification of behavior in *Drosophila* by targeted expression of a temperature-sensitive shibire allele in defined neurons. J. Neurobiol. 47, 81–92. (10.1002/neu.1018) 11291099

[78] Waddell S Armstrong JD Kitamoto T Kaiser K Quinn WG 2000 The amnesiac gene product is expressed in two neurons in the *Drosophila* brain that are critical for memory. Cell 103, 805–813. (10.1016/S0092-8674(00)00183-5) 11114336

[79] Dubnau J Grady L Kitamoto T Tully T 2001 Disruption of neurotransmission in *Drosophila* mushroom body blocks retrieval but not acquisition of memory. Nature 411, 476–480. (10.1038/35078077) 11373680

[80] McGuire SE Le PT Davis RL 2001 The role of *Drosophila* mushroom body signaling in olfactory memory. Science 293, 1330–1333. (10.1126/science.1062622) 11397912

[81] Keene AC Krashes MJ Leung B Bernard JA Waddell S 2006 *Drosophila* dorsal paired medial neurons provide a general mechanism for memory consolidation. Curr. Biol. 16, 1524–1530. (10.1016/j.cub.2006.06.022) 16890528

[82] Krashes MJ Keene AC Leung B Armstrong JD Waddell S 2007 Sequential use of mushroom body neuron subsets during *Drosophila* odor memory processing. Neuron 53, 103–115. (10.1016/j.neuron.2006.11.021) 17196534 PMC1828290

[83] Krashes MJ Waddell S 2008 Rapid consolidation to a radish and protein synthesis-dependent long-term memory after single-session appetitive olfactory conditioning in *Drosophila*. J. Neurosci. 28, 3103–3113. (10.1523/JNEUROSCI.5333-07.2008) 18354013 PMC2516741

[84] Trannoy S Redt-Clouet C Dura JM Preat T 2011 Parallel processing of appetitive short- and long-term memories in *Drosophila*. Curr. Biol. 21, 1647–1653. (10.1016/j.cub.2011.08.032) 21962716

[85] Cervantes-Sandoval I Martin-Pena A Berry JA Davis RL 2013 System-like consolidation of olfactory memories in *Drosophila*. J. Neurosci. 33, 9846–9854. (10.1523/JNEUROSCI.0451-13.2013) 23739981 PMC3733538

[86] Johard HA Enell LE Gustafsson E Trifilieff P Veenstra JA Nassel DR 2008 Intrinsic neurons of *Drosophila* mushroom bodies express short neuropeptide F: relations to extrinsic neurons expressing different neurotransmitters. J. Comp. Neurol. 507, 1479–1496. (10.1002/cne.21636) 18205208

[87] Zemelman BV Lee GA Ng M Miesenbock G 2002 Selective photostimulation of genetically chARGed neurons. Neuron 33, 15–22. (10.1016/S0896-6273(01)00574-8) 11779476

[88] Lima SQ Miesenbock G 2005 Remote control of behavior through genetically targeted photostimulation of neurons. Cell 121, 141–152. (10.1016/j.cell.2005.02.004) 15820685

[89] Nagel G 2003 Channelrhodopsin-2, a directly light-gated cation-selective membrane channel. Proc. Natl Acad. Sci. USA 100, 13 940–13 945. (10.1073/pnas.1936192100) PMC283525 14615590

[90] Boyden ES Zhang F Bamberg E Nagel G Deisseroth K 2005 Millisecond-timescale, genetically targeted optical control of neural activity. Nat. Neurosci. 8, 1263–1268. (10.1038/nn1525) 16116447

[91] Schroll C 2006 Light-induced activation of distinct modulatory neurons triggers appetitive or aversive learning in *Drosophila* larvae. Curr. Biol. 16, 1741–1747. (10.1016/j.cub.2006.07.023) 16950113

[92] Suh GS Ben-Tabou de Leon S Tanimoto H Fiala A Benzer S Anderson DJ 2007 Light activation of an innate olfactory avoidance response in *Drosophila*. Curr. Biol. 17, 905–908. (10.1016/j.cub.2007.04.046) 17493811

[93] Gordon MD Scott K 2009 Motor control in a *Drosophila* taste circuit. Neuron 61, 373–384. (10.1016/j.neuron.2008.12.033) 19217375 PMC2650400

[94] Root CM Masuyama K Green DS Enell LE Nässel DR Lee C-H Wang JW 2008 A presynaptic gain control mechanism fine-tunes olfactory behavior. Neuron 59, 311–321. (10.1016/j.neuron.2008.07.003) 18667158 PMC2539065

[95] Gruntman E Turner GC 2013 Integration of the olfactory code across dendritic claws of single mushroom body neurons. Nat. Neurosci. 16, 1821–1829. (10.1038/nn.3547) 24141312 PMC3908930

[96] Gaudry Q Hong EJ Kain J de Bivort BL Wilson RI 2013 Asymmetric neurotransmitter release enables rapid odour lateralization in *Drosophila*. Nature 493, 424–428. (10.1038/nature11747) 23263180 PMC3590906

[97] Yaksi E Wilson RI 2010 Electrical coupling between olfactory glomeruli. Neuron 67, 1034–1047. (10.1016/j.neuron.2010.08.041) 20869599 PMC2954501

[98] Nagel KI Hong EJ Wilson RI 2015 Synaptic and circuit mechanisms promoting broadband transmission of olfactory stimulus dynamics. Nat. Neurosci. 18, 56–65. (10.1038/nn.3895) 25485755 PMC4289142

[99] Lin JY Knutsen PM Muller A Kleinfeld D Tsien RY 2013 ReaChR: a red-shifted variant of channelrhodopsin enables deep transcranial optogenetic excitation. Nat. Neurosci. 16, 1499–1508. (10.1038/nn.3502) 23995068 PMC3793847

[100] Inagaki HK Jung Y Hoopfer ED Wong AM Mishra N Lin JY Tsien RY Anderson DJ 2014 Optogenetic control of *Drosophila* using a red-shifted channelrhodopsin reveals experience-dependent influences on courtship. Nat. Methods 11, 325–332. (10.1038/nmeth.2765) 24363022 PMC4151318

[101] Owald D Felsenberg J Talbot CB Das G Perisse E Huetteroth W Waddell S 2015 Activity of defined mushroom body output neurons underlies learned olfactory behavior in *Drosophila*. Neuron 86, 417–427. (10.1016/j.neuron.2015.03.025) 25864636 PMC4416108

[102] Klapoetke NC 2014 Independent optical excitation of distinct neural populations. Nat. Methods 11, 338–346. (10.1038/nmeth.2836) 24509633 PMC3943671

[103] Aso Y 2014 Mushroom body output neurons encode valence and guide memory-based action selection in *Drosophila*. Elife 4, e04580 (10.7554/eLife.04580) PMC4273436 25535794

[104] Dawydow A 2014 Channelrhodopsin-2-XXL, a powerful optogenetic tool for low-light applications. Proc. Natl Acad. Sci. USA 111, 13 972–13 977. (10.1073/pnas.1408269111) PMC4183338 25201989

[105] AzimiHashemi N 2014 Synthetic retinal analogues modify the spectral and kinetic characteristics of microbial rhodopsin optogenetic tools. Nat. Commun. 5, 5810 (10.1038/ncomms6810) 25503804

[106] Hamada FN Rosenzweig M Kang K Pulver SR Ghezzi A Jegla TJ Garrity PA 2008 An internal thermal sensor controlling temperature preference in *Drosophila*. Nature 454, 217–220. (10.1038/nature07001) 18548007 PMC2730888

[107] McKemy DD Neuhausser WM Julius D 2002 Identification of a cold receptor reveals a general role for TRP channels in thermosensation. Nature 416, 52–58. (10.1038/nature719) 11882888

[108] Peier AM 2002 A TRP channel that senses cold stimuli and menthol. Cell 108, 705–715. (10.1016/S0092-8674(02)00652-9) 11893340

[109] Parisky KM 2008 PDF cells are a GABA-responsive wake-promoting component of the *Drosophila* sleep circuit. Neuron 60, 672–682. (10.1016/j.neuron.2008.10.042) 19038223 PMC2734413

[110] Donlea JM Thimgan MS Suzuki Y Gottschalk L Shaw PJ 2011 Inducing sleep by remote control facilitates memory consolidation in *Drosophila*. Science 332, 1571–1576. (10.1126/science.1202249) 21700877 PMC4064462

[111] Schwaerzel M Monastirioti M Scholz H Friggi-Grelin F Birman S Heisenberg M 2003 Dopamine and octopamine differentiate between aversive and appetitive olfactory memories in *Drosophila*. J. Neurosci. 23, 10 495–10 502. 10.1523/JNEUROSCI.23-33-10495.2003 PMC6740930 14627633

[112] Aso Y Siwanowicz I Bracker L Ito K Kitamoto T Tanimoto H 2010 Specific dopaminergic neurons for the formation of labile aversive memory. Curr. Biol. 20, 1445–1451. (10.1016/j.cub.2010.06.048) 20637624 PMC2929706

[113] Yamagata N Ichinose T Aso Y Plaçais P-Y Friedrich AB Sima RJ Preat T Rubin GM Tanimoto H 2015 Distinct dopamine neurons mediate reward signals for short- and long-term memories. Proc. Natl Acad. Sci. USA 112, 578–583. (10.1073/pnas.1421930112) 25548178 PMC4299218

[114] Huetteroth W Perisse E Lin S Klappenbach M Burke C Waddell S 2015 Sweet taste and nutrient value subdivide rewarding dopaminergic neurons in *Drosophila*. Curr. Biol. 25, 751–758. (10.1016/j.cub.2015.01.036 25728694 PMC4372253

[115] Zemelman BV Nesnas N Lee GA Miesenbock G 2003 Photochemical gating of heterologous ion channels: remote control over genetically designated populations of neurons. Proc. Natl Acad. Sci. USA 100, 1352–1357. (10.1073/pnas.242738899) 12540832 PMC298776

[116] Peabody NC Pohl JB Diao F Vreede AP Sandstrom DJ Wang H Zelensky PK White BH 2009 Characterization of the decision network for wing expansion in *Drosophila* using targeted expression of the TRPM8 channel. J. Neurosci. 29, 3343–3353. (10.1523/JNEUROSCI.4241-08.2009) 19295141 PMC2717795

[117] Flood TF Gorczyca M White BH Ito K Yoshihara M 2013 A large-scale behavioral screen to identify neurons controlling motor programs in the *Drosophila* brain. G3 (Bethesda) 3, 1629–1637. (10.1534/g3.113.006205) 23934998 PMC3789788

[118] Flood TF Iguchi S Gorczyca M White B Ito K Yoshihara M 2013 A single pair of interneurons commands the *Drosophila* feeding motor program. Nature 499, 83–87. (10.1038/nature12208) 23748445 PMC3727048

[119] Bidaye SS Machacek C Wu Y Dickson BJ 2014 Neuronal control of *Drosophila* walking direction. Science 344, 97–101. (10.1126/science.1249964) 24700860

[120] Seeds AM Ravbar P Chung P Hampel S Midgley FM Mensh BD Simpson JH 2014 A suppression hierarchy among competing motor programs drives sequential grooming in *Drosophila*. Elife 3, e02951 (10.7554/eLife.02951) 25139955 PMC4136539

[121] Hergarden AC Tayler TD Anderson DJ 2012 Allatostatin-A neurons inhibit feeding behavior in adult *Drosophila*. Proc. Natl Acad. Sci. USA 109, 3967–3972. (10.1073/pnas.1200778109) 22345563 PMC3309792

[122] Kohatsu S Koganezawa M Yamamoto D 2011 Female contact activates male-specific interneurons that trigger stereotypic courtship behavior in *Drosophila*. Neuron 69, 498–508. (10.1016/j.neuron.2010.12.017) 21315260

[123] Tayler TD Pacheco DA Hergarden AC Murthy M Anderson DJ 2012 A neuropeptide circuit that coordinates sperm transfer and copulation duration in *Drosophila*. Proc. Natl Acad. Sci. USA 109, 20 697–20 702. (10.1073/pnas.1218246109) PMC3528491 23197833

[124] Asahina K Watanabe K Duistermars BJ Hoopfer E González CR Eyjólfsdóttir EA Perona P Anderson DJ 2014 Tachykinin-expressing neurons control male-specific aggressive arousal in *Drosophila*. Cell 156, 221–235. (10.1016/j.cell.2013.11.045) 24439378 PMC3978814

[125] Vasmer D Pooryasin A Riemensperger T Fiala A 2014 Induction of aversive learning through thermogenetic activation of Kenyon cell ensembles in *Drosophila*. Front. Behav. Neurosci. 8, 174 (10.3389/fnbeh.2014.00174) 24860455 PMC4030157

[126] Herman AM Huang L Murphey DK Garcia I Arenkiel BR 2014 Cell type-specific and time-dependent light exposure contribute to silencing in neurons expressing Channelrhodopsin-2. Elife 3, e01481 (10.7554/eLife.01481) 24473077 PMC3904216

[127] Miyawaki A Griesbeck O Heim R Tsien RY 1999 Dynamic and quantitative Ca^2+^ measurements using improved cameleons. Proc. Natl Acad. Sci. USA 96, 2135–2140. (10.1073/pnas.96.5.2135) 10051607 PMC26749

[128] Kerr R Lev-Ram V Baird G Vincent P Tsien RY Schafer WR 2000 Optical imaging of calcium transients in neurons and pharyngeal muscle of *C. elegans*. Neuron 26, 583–594. (10.1016/S0896-6273(00)81196-4) 10896155

[129] Nakai J Ohkura M Imoto K 2001 A high signal-to-noise Ca^2+^ probe composed of a single green fluorescent protein. Nat. Biotechnol. 19, 137–141. (10.1038/84397) 11175727

[130] Tian L 2009 Imaging neural activity in worms, flies and mice with improved GCaMP calcium indicators. Nat. Methods. 6, 875–881. (10.1038/nmeth.1398) 19898485 PMC2858873

[131] Zhao Y 2011 An expanded palette of genetically encoded Ca^2+^ indicators. Science 333, 1888–1891. (10.1126/science.1208592) 21903779 PMC3560286

[132] Akerboom J 2013 Genetically encoded calcium indicators for multi-color neural activity imaging and combination with optogenetics. Front. Mol. Neurosci. 6, 2 (10.3389/fnmol.2013.00002) 23459413 PMC3586699

[133] Chen TW 2013 Ultrasensitive fluorescent proteins for imaging neuronal activity. Nature 499, 295–300. (10.1038/nature12354) 23868258 PMC3777791

[134] Miesenbock G De Angelis DA Rothman JE 1998 Visualizing secretion and synaptic transmission with pH-sensitive green fluorescent proteins. Nature 394, 192–195. (10.1038/28190) 9671304

[135] Nikolaev VO Bunemann M Hein L Hannawacker A Lohse MJ 2004 Novel single chain cAMP sensors for receptor-induced signal propagation. J. Biol. Chem. 279, 37 215–37 218. (10.1074/jbc.C400302200) 15231839

[136] Jin L Han Z Platisa J Wooltorton JR Cohen LB Pieribone VA 2012 Single action potentials and subthreshold electrical events imaged in neurons with a fluorescent protein voltage probe. Neuron 75, 779–785. (10.1016/j.neuron.2012.06.040) 22958819 PMC3439164

[137] Gong Y Wagner MJ Zhong Li J Schnitzer MJ 2014 Imaging neural spiking in brain tissue using FRET-opsin protein voltage sensors. Nat. Commun. 5, 3674 (10.1038/ncomms4674) 24755708 PMC4247277

[138] Rosay P Armstrong JD Wang Z Kaiser K 2001 Synchronized neural activity in the *Drosophila* memory centers and its modulation by amnesiac. Neuron 30, 759–770. (10.1016/S0896-6273(01)00323-3) 11430809

[139] Fiala A Spall T Diegelmann S Eisermann B Sachse S Devaud J-M Buchner E Galizia CG 2002 Genetically expressed cameleon in *Drosophila melanogaster* is used to visualize olfactory information in projection neurons. Curr. Biol. 12, 1877–1884. (10.1016/S0960-9822(02)01239-3) 12419190

[140] Ng M Roorda RD Lima SQ Zemelman BV Morcillo P Miesenbock G 2002 Transmission of olfactory information between three populations of neurons in the antennal lobe of the fly. Neuron 36, 463–474. (10.1016/S0896-6273(02)00975-3) 12408848

[141] Wang JW Wong AM Flores J Vosshall LB Axel R 2003 Two-photon calcium imaging reveals an odor-evoked map of activity in the fly brain. Cell 112, 271–282. (10.1016/S0092-8674(03)00004-7) 12553914

[142] Yu D Akalal DB Davis RL 2006 *Drosophila* alpha/beta mushroom body neurons form a branch-specific, long-term cellular memory trace after spaced olfactory conditioning. Neuron 52, 845–855. (10.1016/j.neuron.2006.10.030) 17145505 PMC1779901

[143] Wang Y Mamiya A Chiang AS Zhong Y 2008 Imaging of an early memory trace in the *Drosophila* mushroom body. J. Neurosci. 28, 4368–4376. (10.1523/JNEUROSCI.2958-07.2008) 18434515 PMC3413309

[144] Akalal DB Yu D Davis RL 2010 A late-phase, long-term memory trace forms in the gamma neurons of *Drosophila* mushroom bodies after olfactory classical conditioning. J. Neurosci. 30, 16 699–16 708. (10.1523/JNEUROSCI.1882-10.2010) PMC3380342 21148009

[145] Zhang S Roman G 2013 Presynaptic inhibition of gamma lobe neurons is required for olfactory learning in *Drosophila*. Curr. Biol. 23, 2519–2527. (10.1016/j.cub.2013.10.043) 24291093

[146] Yu D Keene AC Srivatsan A Waddell S Davis RL 2005 *Drosophila* DPM neurons form a delayed and branch-specific memory trace after olfactory classical conditioning. Cell 123, 945–957. (10.1016/j.cell.2005.09.037) 16325586

[147] Cervantes-Sandoval I Davis RL 2012 Distinct traces for appetitive versus aversive olfactory memories in DPM neurons of *Drosophila*. Curr. Biol. 22, 1247–1252. (10.1016/j.cub.2012.05.009) 22658595 PMC3396741

[148] Liu X Davis RL 2009 The GABAergic anterior paired lateral neuron suppresses and is suppressed by olfactory learning. Nat. Neurosci. 12, 53–59. (10.1038/nn.2235) 19043409 PMC2680707

[149] Akalal DB Yu D Davis RL 2011 The long-term memory trace formed in the *Drosophila* alpha/beta mushroom body neurons is abolished in long-term memory mutants. J. Neurosci. 31, 5643–5647. (10.1523/JNEUROSCI.3190-10.2011) 21490205 PMC3118425

[150] Wang Y 2004 Stereotyped odor-evoked activity in the mushroom body of *Drosophila* revealed by green fluorescent protein-based Ca^2+^ imaging. J. Neurosci. 24, 6507–6514. (10.1523/JNEUROSCI.3727-03.2004) 15269261 PMC6729867

[151] Honegger KS Campbell RA Turner GC 2011 Cellular-resolution population imaging reveals robust sparse coding in the *Drosophila* mushroom body. J. Neurosci. 31, 11 772–11 785. (10.1523/JNEUROSCI.1099-11.2011) PMC3180869 21849538

[152] Lin AC Bygrave AM de Calignon A Lee T Miesenbock G 2014 Sparse, decorrelated odor coding in the mushroom body enhances learned odor discrimination. Nat. Neurosci. 17, 559–568. (10.1038/nn.3660) 24561998 PMC4000970

[153] Riemensperger T Voller T Stock P Buchner E Fiala A 2005 Punishment prediction by dopaminergic neurons in *Drosophila*. Curr. Biol. 15, 1953–1960. (10.1016/j.cub.2005.09.042) 16271874

[154] Mao Z Davis RL 2009 Eight different types of dopaminergic neurons innervate the *Drosophila* mushroom body neuropil: anatomical and physiological heterogeneity. Front. Neural Circuits 3, 5 (10.3389/neuro.04.005.2009) 19597562 PMC2708966

[155] Placais PY 2012 Slow oscillations in two pairs of dopaminergic neurons gate long-term memory formation in *Drosophila*. Nat. Neurosci. 15, 592–599. (10.1038/nn.3055) 22366756

[156] Berry JA Cervantes-Sandoval I Nicholas EP Davis RL 2012 Dopamine is required for learning and forgetting in *Drosophila*. Neuron 74, 530–542. (10.1016/j.neuron.2012.04.007) 22578504 PMC4083655

[157] Sejourne J 2011 Mushroom body efferent neurons responsible for aversive olfactory memory retrieval in *Drosophila*. Nat. Neurosci. 14, 903–910. (10.1038/nn.2846) 21685917

[158] Placais PY Trannoy S Friedrich AB Tanimoto H Preat T 2013 Two pairs of mushroom body efferent neurons are required for appetitive long-term memory retrieval in *Drosophila*. Cell Rep. 5, 769–780. (10.1016/j.celrep.2013.09.032) 24209748

[159] Pai TP Chen CC Lin HH Chin A-L Lai JS-Y Lee P-T Tully T Chiang A-S 2013 *Drosophila* ORB protein in two mushroom body output neurons is necessary for long-term memory formation. Proc. Natl Acad. Sci. USA 110, 7898–7903. (10.1073/pnas.1216336110) 23610406 PMC3651462

[160] Cao G Platisa J Pieribone VA Raccuglia D Kunst M Nitabach MN 2013 Genetically targeted optical electrophysiology in intact neural circuits. Cell 154, 904–913. (10.1016/j.cell.2013.07.027) 23932121 PMC3874294

[161] Shafer OT Kim DJ Dunbar-Yaffe R Nikolaev VO Lohse MJ Taghert PH 2008 Widespread receptivity to neuropeptide PDF throughout the neuronal circadian clock network of *Drosophila* revealed by real-time cyclic AMP imaging. Neuron 58, 223–237. (10.1016/j.neuron.2008.02.018) 18439407 PMC2586874

[162] Yao Z Shafer OT 2014 The *Drosophila* circadian clock is a variably coupled network of multiple peptidergic units. Science 343, 1516–1520. (10.1126/science.1251285) 24675961 PMC4259399

[163] Zhang J Hupfeld CJ Taylor SS Olefsky JM Tsien RY 2005 Insulin disrupts beta-adrenergic signalling to protein kinase A in adipocytes. Nature 437, 569–573. (10.1038/nature04140) 16177793

[164] Tomchik SM Davis RL 2009 Dynamics of learning-related cAMP signaling and stimulus integration in the *Drosophila* olfactory pathway. Neuron 64, 510–521. (10.1016/j.neuron.2009.09.029) 19945393 PMC4080329

[165] Gervasi N Tchenio P Preat T 2010 PKA dynamics in a *Drosophila* learning center: coincidence detection by rutabaga adenylyl cyclase and spatial regulation by dunce phosphodiesterase. Neuron 65, 516–529. (10.1016/j.neuron.2010.01.014) 20188656

[166] Inagaki HK 2012 Visualizing neuromodulation *in vivo*: TANGO-mapping of dopamine signaling reveals appetite control of sugar sensing. Cell 148, 583–595. (10.1016/j.cell.2011.12.022) 22304923 PMC3295637

[167] Boto T Louis T Jindachomthong K Jalink K Tomchik SM 2014 Dopaminergic modulation of cAMP drives nonlinear plasticity across the *Drosophila* mushroom body lobes. Curr. Biol. 24, 822–831. (10.1016/j.cub.2014.03.021) 24684937 PMC4019670

[168] Klarenbeek JB Goedhart J Hink MA Gadella TW Jalink K 2011 A mTurquoise-based cAMP sensor for both FLIM and ratiometric read-out has improved dynamic range. PLoS ONE 6, e19170 (10.1371/journal.pone.0019170) 21559477 PMC3084777

[169] Allen MD Zhang J 2006 Subcellular dynamics of protein kinase A activity visualized by FRET-based reporters. Biochem. Biophys. Res. Commun. 348, 716–721. (10.1016/j.bbrc.2006.07.136) 16895723

[170] Hu A Zhang W Wang Z 2010 Functional feedback from mushroom bodies to antennal lobes in the *Drosophila* olfactory pathway. Proc. Natl Acad. Sci. USA 107, 10 262–10 267. (10.1073/pnas.0914912107) PMC2890443 20479249

[171] Liu WW Wilson RI 2013 Glutamate is an inhibitory neurotransmitter in the *Drosophila* olfactory system. Proc. Natl Acad. Sci. USA 110, 10 294–10 299. (10.1073/pnas.1220560110) PMC3690841 23729809

[172] Mauss AS Meier M Serbe E Borst A 2014 Optogenetic and pharmacologic dissection of feedforward inhibition in *Drosophila* motion vision. J. Neurosci. 34, 2254–2263. (10.1523/JNEUROSCI.3938-13.2014) 24501364 PMC6608528

[173] Peled ES Newman ZL Isacoff EY 2014 Evoked and spontaneous transmission favored by distinct sets of synapses. Curr. Biol. 24, 484–493. (10.1016/j.cub.2014.01.022) 24560571 PMC4017949

[174] Melom JE Akbergenova Y Gavornik JP Littleton JT 2013 Spontaneous and evoked release are independently regulated at individual active zones. J. Neurosci. 33, 17 253–17 263. (10.1523/JNEUROSCI.3334-13.2013) 24174659 PMC3812501

[175] Ljaschenko D Ehmann N Kittel RJ 2013 Hebbian plasticity guides maturation of glutamate receptor fields *in vivo*. Cell Rep. 3, 1407–1413. (10.1016/j.celrep.2013.04.003) 23643532

[176] Kauwe G Isacoff EY 2013 Rapid feedback regulation of synaptic efficacy during high-frequency activity at the *Drosophila* larval neuromuscular junction. Proc. Natl Acad. Sci. USA 110, 9142–9147. (10.1073/pnas.1221314110) 23674684 PMC3670382

[177] Maimon G Straw AD Dickinson MH 2010 Active flight increases the gain of visual motion processing in *Drosophila*. Nat. Neurosci. 13, 393–399. (10.1038/nn.2492) 20154683

[178] Seelig JD Chiappe ME Lott GK Dutta A Osborne JE Reiser MB Jayaraman V 2010 Two-photon calcium imaging from head-fixed *Drosophila* during optomotor walking behavior. Nat. Methods 7, 535–540. (10.1038/nmeth.1468) 20526346 PMC2945246

